# Generalized bioelectric impedance‐based equations underestimate body fluids in athletes

**DOI:** 10.1111/sms.14033

**Published:** 2021-08-19

**Authors:** Giuseppe Coratella, Francesco Campa, Catarina N. Matias, Stefania Toselli, Josely C. Koury, Angela Andreoli, Lui­s B. Sardinha, Analiza M. Silva

**Affiliations:** ^1^ Department of Biomedical Sciences for Health Università degli Studi di Milano Milan Italy; ^2^ Department for Life Quality Studies Università degli Studi di Bologna Rimini Italy; ^3^ Bettery Life lab, Bettery Lisbon Portugal; ^4^ CIDEFES ‐Universidade Lusófona Lisboa Portugal; ^5^ Departments of Biomedical and Neuromotor Sciences University of Bologna Bologna Italy; ^6^ Department of Basic and Experimental Nutrition Nutrition Institute State University of Rio de Janeiro Rio de Janeiro Brazil; ^7^ Department of Systems Medicine University of Tor Vergata Rome Italy; ^8^ Exercise and Health Laboratory CIPER Faculdade de Motricidade Humana Universidade de Lisboa Cruz‐Quebrada Portugal

**Keywords:** BIA, body composition, extracellular water, predictive equations, resistance training, total body water

## Abstract

The current study aimed: (i) to external validate total body water (TBW) and extracellular water (ECW) derived from athlete and non‐athlete predictive equations using radioisotope dilution techniques as a reference criterion in male and female athletes; (ii) in a larger sample, to determine the agreement between specific and generalized equations when estimating body fluids in male and female athletes practicing different sports. A total of 1371 athletes (men: *n =* 921, age 23.9 ± 1.4 y; women: *n =* 450, age 27.3 ± 6.8 y) participated in this study. All athletes underwent bioelectrical impedance analyses, while TBW and ECW were assessed with dilution techniques in a subgroup of 185 participants (men: *n =* 132, age 21.7 ± 5.1 y; women: *n =* 53, age 20.3 ± 4.5 y). Two specific and eight generalized predictive equations were tested. Compared to the criterion methods, no mean bias was observed using the athlete‐specific equations for TBW and ECW (−0.32 to 0.05, *p *> 0.05) and the coefficient of determination ranged from *R* ^2^ = 0.83 to 0.94. The majority of the generalized predictive equations underestimated TBW and ECW (*p *< 0.05); *R* ^2^ ranged from 0.66 to 0.89. In the larger sample, all the generalized equations showed lower TBW and ECW values (ranging from −6.58 to −0.19, *p *< 0.05) than specific predictive equations; except for TBW in female power/velocity (one equation) athletes and team sport (two equations). The use of generalized BIA‐based equations leads to an underestimation of TBW, and ECW compared to athlete‐specific predictive equations. Additionally, the larger sample indicates that generalized equations overall provided lower TBW and ECW compared to the athlete‐specific equations.

## 1 INTRODUCTION

The study of body composition in athletes has attracted the interest of researchers and practitioners over the years, given the implications on sports performance and health.^1^, ^2^ By monitoring body composition parameters, the effects of a diet or training could be qualitatively investigated.^1^, ^2^ Considering the different nature of body composition elements that make up the total body mass, different parameters can be measured or estimated.^2^ However, reference methods for assessing body composition are often not available in the practice, so a number of alternative procedures have been implemented.^1^, ^3^

Among the possible methods, the bioelectrical impedance analysis (BIA) represents a portable, user‐friendly, and low‐cost tool that makes it possible to estimate a wide range of parameters, including total body water (TBW) and extracellular water (ECW).^1^, ^3^ In particular, TBW represents the major component of body mass and its unrestored loss reflects dehydration, a condition that negatively affects performance and health.^4^, ^5^, ^6^ In addition, the distribution of the fluids between intra‐ and extra‐cellular compartments provides information about the body cell mass, the metabolically active portion of body mass, and fluid retention and inflammation.^7^ Due to the association between the body fluids and the bioelectrical properties,^8^, ^9^, ^10^ BIA represents a valid alternative to the gold standard methods identified as the dilution techniques.^3^, ^4^

The BIA provides raw bioelectrical values that can be inserted into predictive equations for estimating total TBW and ECW.^1^, ^3^ Most of the predictive equations have been developed and validated in the general population,^11^, ^12^, ^13^, ^14^, ^15^, ^16^ but the extent to which athletes water compartments may have been incorrectly estimated is still to be determined. Indeed, specific predictive equations for assessing TBW and ECW in athletes have recently been provided^17^ and used in some studies.^18^, ^19^ In this regard, previous publications reported that BIA‐based prediction equations yield inaccurate body composition estimates when applied in samples that differ from the original derivation sample.^3^ This may be due to the specific body composition features that characterize each population. For example, athletes show a greater phase angle and therefore a higher intracellular water/ECW ratio compared to the general population.^1^, ^3^ Therefore, predictive models may not be particularly accurate if applied to samples with characteristics that are far from those of the sample on which they were developed. Similarly, given that several BIA devices may show a lack of agreement in the measured raw bioelectrical values,^3^ in order to achieve a greater accuracy each equation should be applied with devices similar to those used in their development.

Notwithstanding, there are still studies being published that used generalized equations,^20^, ^21^, ^22^ as well as those that used manufacturer‐provided proprietary predictive formulas.^23^, ^24^, ^25^, ^26^, ^27^ Some researchers have warned against the use of generalized equations in athletes, since inaccurate output could be extrapolated.^1^, ^3^, ^28^ However, the magnitude of the possible bias compared with the dilution techniques as criterion, as well as its direction, has not been determined thus far. Additionally, a comparison between TBW and ECW estimated specific versus generalized equations in athletes practicing different sports has not been performed yet. This may help to quantify the agreement between using specific and generalized estimations. Therefore, the aims of the present study were as follows: (i) to external validate total body water and extracellular water derived from specific and generalized predictive equations using dilution techniques as the reference criterion in male and female athletes; (ii) to determine the agreement between specific and generalized equations when estimating body fluids in male and female athletes practicing different sports, in a larger sample. Since athletes may show different body composition features compared to the general population, our hypothesis was that bioelectrical impedance prediction models derived from non‐athletes would result in different TBW and ECW values compared with criterion methods and specific equations developed for adult athletes.

## 2 METHODS

### 2.1 Subjects

This was a cross‐sectional, observational study on 1371 (70% Caucasians and 30% Hispanics) athletes (men: *n =* 921, age 23.9 ± 1.4 y, BMI 23.8 ± 6.4 kg/m^2^; women: *n =* 450, age 27.3 ± 6.8 y, BMI 21.9 ± 2.2 kg/m^2^) involved in different sport modalities who were sorted out into 3 groups: endurance (cycling, marathon, pentathlon, cross country skiing, long distance rowing, and triathlon), velocity/power (athletics including jumpers, throwers, and short distance runners, badminton, boxing, CrossFit, judo, karate, kickboxing, rowing, rhythmic gymnastics, swimming including short distance swimmers, and tennis), and team sports (basketball, field hockey, handball, rugby, soccer, volleyball, and water polo). In order to address the objectives of the present investigation, a validation and an agreement study were conducted. All the athletes were initially subjected to BIA and involved into the agreement study, while a subgroup of 185 athletes (men: *n =* 132, age 21.7 ± 5.1 y, BMI 22.8 ± 2.6 kg/m^2^; women: *n =* 53, age 20.3 ± 4.5 y, BMI 21.7 ± 2.0 kg/m^2^) practicing different sports (athletics, basketball, handball, judo, karate, pentathlon, rugby, soccer, swimming, triathlon, and volleyball) were involved into the validation study.

The following inclusion criteria were used: (1) 10 or more hours of training per week, (2) negative test outcomes for performance‐enhancing drugs, and (3) not taking any medications. All subjects were informed about the possible risks of the investigation before giving written informed consent to participate. All procedures were approved by the bioethics committee of the University of Bologna and were conducted in accordance with the declaration of Helsinki for human studies (Ethical Approval Code: 25027).

### 2.2 Procedures

Participants came to the laboratory refraining and alcohol or stimulant beverages and fasting for at least 3 h. Testing began promptly at 08:00 with at least 15 h from the last exercise session.

Body weight was measured with a scale without shoes and wearing minimal clothes, to the nearest 0.01 kg and height was measured to the nearest 0.1 cm with a stadiometer (Seca).

The impedance measurements were performed with a BIA analyzer (BIA‐101, RJL/Akern Systems) using an electric current at a frequency of 50 kHz. Measurements were made on an isolated cot from electrical conductors, the subjects were in the supine position with a leg opening of 45° compared to the median line of the body and the upper limbs, distant 30° from the trunk. After cleansing the skin with alcohol, two electrodes (Biatrodes Akern Srl) were placed on the right hand back and two electrodes (Biatrodes Akern Srl) on the corresponding foot. Prior to each test, the analyzer was calibrated with the calibration deemed successful if R value is 383 Ohm and Xc equal to 46 Ohm. The test‐retest CV in 10 participants in our laboratory for R and Xc is 0.3% and 0.9%, respectively. The selected predictive equations for TBW and ECW estimations are shown in Table 1.

**TABLE 1:** Predictive bioelectrical impedance‐based equations for body composition estimation using a foot‐to‐hand device at a sampling frequency of 50 kHz in healthy adults

Author	Equation	Sample	BIA device	Age (Years)	*R* ^2^	SEE	Validation on athletes
Total body water (L)							
Matias et al.^17^	0.286 + 0.195*S^2^/R + 0.385*Wt + 5.086*sex^a^	Men and women athletes (*N =* 208)	BIA‐101, RJL/Akern	μ 21	0.93	2.42 kg	Yes
Sun et al.^11^	Male: 1.20 + 0.45*S^2^R + 0.18*Wt Female: 3.75 + 0.45* S^2^R + 0.11*Wt	Whites and blacks of both sexes (*N =* 1801)	BIA‐101, RJL	12–94	0.84 0.79	3.80 L 2.60 L	No No
Schoeller et al.^16^	0.499* S^2^R + 0.080 S^2^R*Wt + 2.9	Caucasians and African Americans of both sexes (*N =* 125)	BIA‐101, RJL and Xitron 4000B	14–53	0.93	2.50 kg	No
Kushner et al.^15^	0.59* S^2^R + 0.065*Wt + 0.04	From neonates to adults of both sexes (*N =* 175)	BIA‐101, RJL	0.02–67	0.99	1.41 kg	No
Kotler et al.^12^	Male: 0.58* (S^1.62^/Z^0.7^)* (1/1.35) + 0.32*Wt − 3.66 Female: 0.76*(S^1.99^/Z^0.58^)* (1/18.91) + 0.14*Wt − 0.86	White, black, and Hispanic men and women (*N =* 332)	BIA‐101, RJL	μ 41	0.86 0.82	7.60 % 8.20 %	No No
Lukaski et al.^14^	0.377* S^2^/R + 0.14*Wt − 0.08*age + 2.9*sex^a^ + 4.65	Caucasians and African Americans of both sexes (*N =* 110)	BIA‐101, RJL	20–73	0.99	1.41 kg	No
Extracellular water (L)							
Matias et al.^17^	1.579 + 0.055* S^2^/R + 0.127*Wt + 0.006* S^2^/Xc + 0.932*sex^a^	Men and women athletes (*N =* 208)	BIA‐101, RJL/Akern	μ 21	0.84	1.33 kg	Yes
Sergi et al.^13^	−5.22 + 0.2* S^2^/R + 0.005*S^2^/Xc + 0.08*Wt + 1.9 + 1.86*sex^b^	Caucasians of both sexes (*N =* 40)	BIA‐101, RJL/Akern	21–81	0.89	1.70 L	No
Lukaski et al.^14^	0.189*(S^2^/R) + 0.052*Wt − 0.0002*(S^2^/Xc) + 1.03	Caucasians and African Americans of both sexes (*N =* 110)	BIA‐101, RJL	20–73	0.88	1.01 L	No

Abbreviations: BIA, bioelectrical impedance analysis; R, resistance (ohm); R^2^, coefficient of determination; S, Stature (cm); SEE, standard error of estimation. Wt, body mass (kg); Xc, reactance (ohm); Z, impedance (ohm).

^a^ 0 if female; 1 if male.

^b^ 1 if female; 0 if male.

Matias et al.^17^ Sun et al.^11^ Schoeller et al.^16^ Kushner et al.^15^ Kotler et al.^12^ and Lukaski et al.^14^ predictive equations were validated using deuterium dilution; whereas Matias et al.^17^ Sergi et al.^13^ and Lukaski et al.^14^ were validated using bromide dilution. Only Matias et al.^17^ predictive equations were validated in athletes. The equations used were chosen because of their popularity and as being representative of the many equations that have been published.^29^

Following the collection of a baseline urine sample, each participant was given an oral dose of 0.1 g of 99.9%^2^H_2_O per kg of body weight (Sigma‐Aldrich) for the determination of total body water by deuterium dilution using a Hydra stable isotope ratio mass spectrometer (PDZ, Europa Scientific, UK). Subjects were encouraged to void their bladder prior to the 4‐h equilibration period and subsequent sample collection, due to inadequate mixing of pre‐existing urine in the bladder. Urine samples were prepared for^1^H/^2^H analyses using the equilibration technique by Prosser and Scrimgeour.^30^

Extracellular water was assessed from the sodium bromide (NaBr) dilution method after the subject consumed 0.030 g of 99.0% NaBr (Sigma‐Aldrich) per kg of body weight, diluted in 50 ml of distilled‐deionized water. Baseline samples of saliva were collected before sodium bromide oral dose administration, and enriched samples were collected 3 h post‐dose administration.

### 2.3 Statistical analysis

Data were analyzed with SPSS v. 27.0 (SPSS, IBM Corp.,) and MedCalc Statistical Software v.11.1.1.0, 2009 (Mariakerke, Belgium). The Shapiro‐Wilk test was used to check the normal distribution of data. Sphericity of the data was preliminary assessed using the Mauchly's test. To external validate the selected equations, the resulting TBW and ECW were validated against the same parameters assessed using the reference method. A paired sample t test was employed to compare the mean values obtained from the reference technique and from BIA. Linear regression analysis was performed considering the values obtained from reference methods as dependent variables and the estimated parameters as independent variables. Agreement between specific and generalized predictive equations in the larger sample of athletes sorted out by sports modality was determined using the Bland‐Altman method, Lin's concordance correlation coefficient (CCC), including precision (ρ) and accuracy (C_b_) indexes, and by McBride's ^31^ strength concordance (almost perfect>0.99; substantial>0.95 to 0.99; moderate=0.90–0.95; and poor<0.90).

## 3 RESULTS

### 3.1 External‐validation study

In men, with the exception of the equation by Matias specific predictive equations, all other generalized predictive equations showed a significant difference (*p *< 0.05) in TBW estimation as compared with the deuterium dilution, as shown in Table 2. The extracellular water estimated by Sergi predictive equation differed with respect to the reference method. For athletic women, Matias et al.^17^ and Kotler et al.^12^ predictive equations did not present differences when compared with TBW values obtained using radioisotope dilution method. However, only Matias et al.^17^ predictive equations did not present differences when compared with ECW values obtained using radioisotope dilution method.

**TABLE 2:** Validation of the regression equations in athletes

	Mean ± SD	Regression analysis		CCC analysis			Agreement analysis		
		*R* ^2^	SEE (kg)	CCC	*ρ*	C_b_	Bias	95% LoA	Trend
Men (*n *= 132)									
Total body water (L)									
Deuterium	49.5 ± 7.4	‐	‐	‐	‐	‐	‐	‐	‐
Matias et al. (2016)	49.6 ± 7.2	0.91	2.14	0.957	0.958	0.999	0.05	−4.14; 4.25	*r = *−0.149; *p =* 0.089
Sun et al. (2013)	47.7 ± 6.8*	0.87	2.62	0.903	0.937	0.963	−1.80*	−6.94; 3.33	*r = *−0.282; *p =* 0.001
Schoeller et al. (2000)	45.4 ± 6.3*	0.87	2.68	0.783	0.934	0.838	−4.06*	−9.48; 1.37	*r = *−0.451; p<0.001
Kushner et al. (1992)	48.1 ± 7.1*	0.86	2.73	0.912	0.931	0.979	−1.43*	−6.78; 3.92	*r = *−0.143; *p =* 0.101
Kotler et al. (1990)	47.9 ± 6.5*	0.86	2.76	0.895	0.930	0.962	−1.63*	−7.12; 3.86	*r = *−0.379; *p <* 0.001
Lukaski et al. (1988)	44.1 ± 5.5*	0.88	2.58	0.668	0.939	0.712	−5.44*	−11.26;0.38	*r = *0.663; *p <* 0.001
Extracellular water (L)									
Bromide	19.2 ± 2.9	‐	‐	‐	‐	‐	‐	‐	‐
Matias et al. (2016)	19.2 ± 2.8	0.84	1.12	0.914	0.916	0.997	0.05	−2.29; 2.39	*r = *−0.155; *p =* 0.076
Sergi et al. (1994)	17.4 ± 3.0*	0.66	1.75	0.692	0.811	0.854	−1.75*	−5.36; 1.86	*r = *0.013; *p =* 0.878
Lukaski et al. (1988)	19.1 ± 2.7	0.66	1.73	0.812	0.815	0.996	−0.06	−3.50; 3.38	*r = *−0.145; *p =* 0.097
Women (*n* = 53)									
Total body water (L)									
Deuterium	35.88 ± 5.3	‐	‐	‐	‐	‐	‐	‐	‐
Matias et al. (2016)	35.6 ± 5.3	0.94	1.35	0.966	0.967	0.998	−0.28	−2.91; 2.38	*r = *−0.001; *p =* 0.995
Sun et al. (2013)	34.4 ± 4.3*	0.89	1.76	0.883	0.942	0.937	−1.47*	−5.13; 2.19	*r = *−0.507; *p <* 0.001
Schoeller et al. (2000)	34.2 ± 4.5*	0.88	1.82	0.876	0.938	0.933	−1.65*	−5.33; 2.03	*r = *−0.424; *p <* 0.001
Kushner et al. (1992)	35.2 ± 5.1*	0.88	1.83	0.927	0.935	0.991	−0.67*	−4.33; 2.98	*r = *−0.098; *p =* 0.485
Kotler et al. (1990)	36.4 ± 4.4	0.87	1.88	0.911	0.933	0.977	0.53	−3.35; 4.41	*r = *−0.460; *p =* 0.001
Lukaski et al. (1988)	31.75 ± 5.3*	0.89	1.75	0.648	0.944	0.686	−4.13*	−8.05; −0.21	*r = *−0.664; *p <* 0.001
Extracellular water (L)									
Bromide	14.6 ± 1.9	‐	‐	‐	‐	‐	‐	‐	‐
Matias et al. (2016)	14.8 ± 1.8	0.83	0.80	0.904	0.909	0.994	−0.32	−1.44; 1.69	*r = *−0.191; *p =* 0.170
Sergi et al. (1994)	12.3 ± 2.2*	0.84	0.78	0.592	0.856	0.664	−2.34*	−4.02; −0.65	*r = *0.268; *p =* 0.052
Lukaski et al. (1988)	12.7 ± 1.8*	0.77	0.93	0.585	0.894	0.654	−1.89*	−3.65; −0.12	*r = *−0.147; *p =* 0.295

Note

*r* ^2^, coefficient of correlation. *= Significant differences with the reference method (*p *< 0.05).

Abbreviations: C_b_, accuracy; CCC, concordance correlation coefficient; CV, coefficient of variation; DXA, dual‐energy x‐ray absorptiometry; LoA, limits of agreement; SEE, standard error of estimation; *ρ*, precision.

Total body water estimation using specific or generalized equations was highly correlated (*R* ^2^ ranged from 0.86 to 0.94) with the reference values in both sexes with the highest coefficient of determination observed using the model developed for athletes (Matias et al.^17^) (Table 2). For the ECW, an *R* ^2^ value lower than 0.80 was found for the predictive equations developed by Sergi et al.^13^ in men and Lukaski et al.^14^ for men and women while a coefficient of determination of 84% was found using the specific models developed by Matias et al.^17^ (Table 2).

Concerning the concordance analysis, the best performance was observed for Matias et al.^17^ predictive equation, in both men and women, with a concordance correlation coefficient of 0.957 and 0.966 (considered as substantial by McBride^31^), a precision of 0.958 and 0.967, and an accuracy of 0.999 and 0.998, respectively. Similar results were observed on the concordance analysis for ECW, with an observed concordance correlation coefficient and precision higher than 0.90, and an accuracy higher the 0.99 for both men and women using Matias et al.^17^ predictive equation (Table 2).

For the agreement analysis performed for TBW assessment, no trend was observed in Matias and Kushner equations, while a trend (*p *< 0.05) was verified between the mean and the difference of methods for the Sun, Schoeller, Kotler, and Lukaski equations for both men and women as shown in Table 2. No trend was observed for extracellular water for any predictive equations, in men or women, as shown in Table 2.

### 3.2 Comparison study

Considering Matias et al.^17^ predictive equation as the one specifically developed for an athlete´s population, other equations presented in the literature were scrutinized in order to verify their agreement with this method. In the male sample, all equations showed significant lower values of body water compartments (TBW and ECW) compared to the specific equation (*p *< 0.05), with the exception of Kushner et al.^15^ equation in the velocity/power male athletes. Additionally, a trend between the mean and the difference of the methods in assessing TBW and ECW was observed in all the agreement analysis, with the exception observed using he unspecified TBW models developed by Schoeller (across the sports categories) and Kotler et al.^12^ (velocity/power athletes). This trend observed in TBW and ECW estimation means that the generalized equations tend to under and overestimate TBW depending on the magnitude of the water compartments.

For the female subsample, differences between the specific and generalized equations were observed for all comparisons, with the exception of Kotler et al.^12^ equation in team sports players and Sun et al.^11^ predictive equation in both team sports and velocity/power athletes. Additionally, a trend between the mean and the difference of the equations used to determine TBW and ECW was observed in all the agreement analysis, with the exception of the models for predicting TBW developed by Lukaski et al.^14^ (endurance sports and velocity/power athletes) and by Sun et al.^11^ Schoeller et al.^16^ and Kotler et al.^12^ in velocity/power athletes, as shown in Table 3. The predictions of TBW and ECW using the unspecified models tend to be exacerbated in the athletes showing lower levels of body water.

**TABLE 3:** Agreement analysis between specific and unspecific equations

	Endurance (*n* = 151)				Team sports (*n *= 516)				Velocity/power (*n* = 254)			
	Mean ± SD	Bias	95% LoA	Trend	Mean ± SD	Bias	95% LoA	Trend	Mean ± SD	Bias	95% LoA	Trend
Men (*n* = 921)												
Total body water (L)												
Matias et al. (2016)	45.3 ± 4.4	‐	‐	‐	53.1 ± 7.4	‐	‐	‐	48.5 ± 5.3	‐	‐	‐
Sun et al. (2013)	43.9 ± 4.9*	−1.41	−4.56; 1.74	*r = *−0.291; p<0.001	51.5 ± 7.8*	−1.66	−5.58; 2.26	*r = *−0.186; *p <* 0.001	47.7 ± 5.8*	−0.79	−4.35; 2.77	*r = *−0.269; *p <* 0.001
Schoeller et al. (2000)	41.8 ± 4.6*	−3.41	−7.44; 0.62	*r = *−0.116; *p =* 0.156	48.5 ± 7.2*	−4.60	−9.91; 0.61	*r = *0.077; *p =* 0.080	45.5 ± 5.5*	−2.98	−7.56; 1.60	*r = *−0.071; *p =* 0.256
Kushner et al. (1992)	44.0 ± 5.3*	−1.23	−6.14; 3.68	*r = *−0.370; *p <* 0.001	51.4 ± 8.2*	−1.65	−7.09; 4.60	*r = *−0.247; *p <* 0.001	48.2 ± 6.3	−0.28	−5.80; 5.24	*r = *−0.343; *p <* 0.001
Kotler et al. (1990)	43.9 ± 4.5*	−1.40	−2.39; −0.40	*r = *−0.228; *p =* 0.005	51.8 ± 7.5*	−1.33	−2.74; 0.01	*r = *−0.104; *p =* 0.018	47.3 ± 5.4*	−1.24	−2.16; −0.31	*r = *−0.072; *p =* 0.254
Lukaski et al. (1988)	40.6 ± 4.1*	−4.66	−7.90; −1.39	*r = *0.211; *p =* 0.009	46.9 ± 6.2*	−6.24	−10.86; −2.61	*r = *0.523; *p <* 0.001	43.6 ± 4.6*	−4.84	−8.58; −1.09	*r = *0.376; *p <* 0.001
Extracellular water (L)												
Matias et al. (2016)	18.1 ± 1.7	‐	‐	‐	21.1 ± 2.9	‐	‐	‐	19.4 ± 2.1	‐	‐	‐
Sergi et al. (1994)	15.6 ± 2.2*	−2.45	−4.01; −0.84	*r = *−0.602; *p <* 0.001	19.0 ± 3.5*	−2.03	−6.65; 2.59	*r = *−0.524; *p <* 0.001	17.3 ± 2.6*	−2.01	−3.62; −0.37	*r = *−0.565; *p =* 0.001
Lukaski et al. (1988)	17.6 ± 2.0*	−0.53	−2.48; 1.37	*r = *−0.394; *p <* 0.001	20.4 ± 3.2*	−0.63	−2.78; 1.50	*r = *−0.272; *p <* 0.001	19.2 ± 2.4*	−0.19	−2.07; 1.69	*r = *−0.329; *p <* 0.001

	Endurance (*n* = 76)				Team sports (*n* = 197)				Velocity/power (*n* = 177)			
	Mean ± SD	Bias	95% LoA	Trend	Mean ± SD	Bias	95% LoA	Trend	Mean ± SD	Bias	95% LoA	Trend
Women (*n *= 450)												
Total body water (L)												
Matias et al. (2016)	36.7 ± 3.2		‐	‐	40.9 ± 3.7	‐	‐	‐	37.3 ± 4.7	‐	‐	‐
Sun et al. (2013)	35.1 ± 4.7*	−1.67	−10.33; 6.99	*r = *−0.397; *p <* 0.001	40.8 ± 5.1	−0.15	−9.67; 9.37	*r = *−0.336; *p <* 0.001	37.0 ± 5.1	−0.39	−10.99; 10.24	*r = *−0.105; *p =* 0.239
Schoeller et al. (2000)	33.1 ± 4.5*	−3.75	−12.35; 5.17	*r = *−0.356; *p =* 0.001	38.4 ± 4.9*	−2.53	−11.70; 6.64	*r = *−0.299; *p <* 0.001	36.4 ± 5.6*	−2.17	−12.46; 8.12	*r = *−0.052; *p =* 0.560
Kushner et al. (1992)	33.9 ± 5.2*	−2.75	−12.62; 7.12	*r = *−0.391; *p =* 0.001	39.9 ± 5.6*	−0.99	−11.18; 9.20	*r = *−0.429; *p <* 0.001	36.4 ± 4.9*	−0.99	−12.15; 10.19	*r = *−0.196; *p =* 0.027
Kotler et al. (1990)	35.3 ± 4.2*	−1.46	−9.47; 6.55	*r = *−0.300; *p =* 0.012	40.8 ± 4.5	−0.19	−9.00; 8.64	*r = *−0.221; *p =* 0.001	36.3 ± 4.2*	−1.02	−11.99; 9.65	*r = *0.100; *p =* 0.264
Lukaski et al. (1988)	30.2 ± 3.9*	−6.58	−13.98; 0.82	*r = *−0.233; *p =* 0.052	34.9 ± 3.9*	−6.05	−14.16; 2.06	*r = *−0.073; *p =* 0.030	31.7 ± 4.3*	−5.62	−15.26; 4.02	*r = *0.099; *p =* 0.270
Extracellular water (L)												
Matias et al. (2016)	14.7 ± 1.6	‐	‐	‐	16.8 ± 1.8	‐	‐	‐	15.3 ± 1.9	‐	‐	‐
Sergi et al. (1994)	11.7 ± 2.1*	−2.97	−4.54; −1.34	*r = *−0.575; *p <* 0.001	14.3 ± 2.3*	−2.50	−4.06; −0.93	*r = *−0.576; *p <* 0.001	12.6 ± 2.3*	−2.72	−4.17; 1.26	*r = *−0.434; *p <* 0.001
Lukaski et al. (1988)	13.2 ± 1.9*	−0.93	−2.90; 1.04	*r = *−0.379; *p =* 0.001	16.1 ± 2.2*	−0.70	−2.48; 1.08	*r = *−0.405; *p <* 0.001	14.7 ± 2.0*	−0.60	−2.38; 1.18	*r = *−0.265; *p =* 0.003

Note

*Significant differences with the specific equation (*p *< 0.05).

Abbreviation: LoA, limits of agreement.

## 4 DISCUSSION

The overall intentions of the present investigation were as follows: (i) to external validate TBW and ECW obtained using dilution techniques as criterion with those estimated from specific and generalized BIA‐based equations in male and female athletes; (ii) to determine the agreement between specific and generalized equations in a larger athletic sample, when estimating body fluids in male and female athletes engaged in endurance, team, and strength/power sports. As hypothesized, generalized equations resulted in less accurate estimations of TBW and ECW compared with the dilution techniques. Additionally, most of the generalized predictive models showed different results when compared with the specific models for athletes.

The present findings showed that only the specific Matias et al.^17^ predictive equation agreed with the values obtained using the criterion, while all the generalized equations underestimated TBW in male and female athletes, with the exception of the Kotler et al.^12^ predictive equation that showed no difference when applied to women. Considering extracellular water, the Sergi et al.^13^ predictive equation underestimated the values obtained with bromide dilution in both men and women, while the predictive model proposed by Lukaski et al.^14^ underestimated extracellular water in women. Furthermore, all the non‐specific equations showed lower body fluid values in comparison with those obtained with the Matias et al.^17^ predictive equations, irrespective of sex and sport. The current outcomes suggest that previous studies using generalized equations have underestimated body fluids in male and female athletes. When aiming to sports‐specific body composition reference values, the monitoring through generalized BIA‐based generalized equations may thus lead to inaccurate estimations.

Precision and accuracy between the selected equations and the reference methods were analyzed with the concordance correlation coefficient analysis, while the Bland‐Altman's analysis was used to determine agreement between methods. A substantial strength of agreement between the Matias et al.^17^ predictive equations and the reference methods was observed in estimating TBW and ECW, while a weaker agreement was found between the other equations with the dilution techniques results. Although no significant trend was observed in Matias et al.^17^ predictive equation for both men and women, the 95% confidence intervals were larger for men. In this regard, total body water could be over‐ or underestimated by ~4.2 kg in men and by ~2.5 kg in women, while extracellular water could be over‐ or underestimated by ~2.3 kg in men and by ~1.5 kg in women. More specifically, considering equation comparison with deuterium dilution in men, Matias et al.^17^ predictive equation explained 91% of the TBW variability, with the lower SEE observed, and being the only equation without differences from the reference method. The Lukaski et al.^14^ predictive equation showed an 88% power explanation of the TBW and a 2.58 kg of SEE while Sun et al.^11^ and Schoeller et al.^16^ predictive equations both presented only 87% explanation of the TBW content regarding the reference method and a 2.60 kg of SEE; Kushner et al.^15^ and Kotler et al.^12^ predictive equations both presented an 86% explanation power and a ~2.kg of SEE. Similar results were observed in the female sample, with Matias et al.^17^ equation explaining 94% of the variability of the TBW assessment (and an SEE of 1.35 kg), followed by Lukaski et al.^14^ and Sun et al.^11^ predictive equations (89% power explanation of the TBW content and ~1.75 kg of SEE), after that Schoeller et al.^16^ and Kushner et al.^15^ predictive equations (88% variability explanation and ~1.82 kg of SEE), and finally, Kotler et al.^12^ predictive equation (87% explanation of the TBW variability with an SEE of 1.88 kg). It is important to note that the generalized models for TBW assessment were obtained in samples of adult non‐sportive people.

In male athletes, Matias equation explained 84% of the total variability of the ECW compartment obtained by the bromide dilution method, while Sergi and Lukaski equations only explained 66% with a higher SEE (~1.7 kg). Regarding the female sample, Matias equation showed no differences from the reference method, explaining 83% of the extracellular compartment (with an SEE of 0.8kg). Despite the Sergi equation explained 84% of total variability of the reference ECW values with a lower SEE (0.78 kg), a significant ECW underestimated was observed. Lukaski equation presented the lowest variability power explanation (77%) and the higher SEE (0.93 kg). It should be highlighted that the Sergi and Lukaski models were developed in a sample of Caucasian and Caucasian and African American non‐sportive people, respectively.

Recognizing the better performance of Matias et al.^17^ equations in estimating the reference TBW and ECW in athletes, the second aim of the current study was to examine how generalized equations agree with the predictive models developed by Matias and collaborators.^17^ In men, all the generalized equations underestimate total body water and extracellular water in endurance and team sports athletes. Regarding the velocity/power group, although Kushner et al.^15^ predictive equation did not show a significant bias, an underestimation and overestimation were observed in athletes with the lower and higher TBW values, respectively. In women, all the generalized equations underestimated TBW and ECW in endurance and power/velocity athletes. Regarding team sports athletes, although the Kotler et al.^12^ predictive equation did not show a significant bias, again a significant trend was found. Taken together these observations indicate that in general, generalized equations underestimated total body and extracellular in athletes, regardless of the sex and the sports categories. It should be also noted that athletes may have different body composition features compared with the general population,^1^, ^3^ so that possible discrepancies in predicted TBW and ECW values between athletes and non–athlete‐derived models may occur when using BIA in athletes.

The current study presents limitations that should be addressed. First, our results are not generalizable to adolescent or senior athletes, since their body composition is overall different from the ones used to elaborate the predictive equations examined here.^32^ Second, our outcomes derive from the use of a foot‐to‐hand technology and a 50 kHz sampling frequency. Therefore, the current findings cannot be extended to different technologies (e.g., BIA in standing position) and sampling frequencies. Last, the present study was conceived as a cross‐sectional investigation and did not assess the ability of any equation to identify the longitudinal training‐induced changes in body fluids.

In conclusion, the specific Matias et al.^17^ equations resulted in valid TBW and ECW estimation when compared to dilution techniques while the generalized equations underestimate body fluids in male and female athletes. Additionally, using a larger sample of athletes engaged in endurance, team and strength and power sports, most of the generalized equations underestimated body fluids when compared to the specific models proposed by Matias et al.^17^ regardless of sex and sports.

### 4.1 Perspectives

The present findings have interesting perspectives. In first instance, data derived from BIA are used to assess body composition in athletes, so that specific values may be assured for a given athlete over the training process. As such, referring to generalized equations may result in inaccurate evaluations. This is not trifling, since many studies used generalized equations to estimate body fluids in athletes or still use generalized equations after the models developed by Matias et al.^17^ have been published.^20^, ^21^, ^22^, ^33^ Furthermore, there is now a wide range of commercial BIA devices, used in research articles, that do not provide information on the equation used for measuring body fluids in athletes.^23^, ^24^, ^27^, ^34^ In this regard, it is important to consider that BIA‐based equations should be applied using raw bioelectrical parameters obtained with devices and sampling frequencies similar to those with which they were developed.^35^ In fact, numerous studies show how different outcomes are obtained using different devices and sampling frequencies.^35^, ^36^ These inaccuracies in assessing body fluids at the group and particularly the individual level may compromise an adequate assessment and monitoring of body fluids over the competitive season. Therefore, caution should be applied when interpreting data extracted from generalized equations or technologies.

## Data Availability

The data that support the findings of this study are available from the corresponding author upon reasonable request.
